# Contrasting Effects of Systemic Monocyte/Macrophage and CD4^+^ T Cell Depletion in a Reversible Ureteral Obstruction Mouse Model of Chronic Kidney Disease

**DOI:** 10.1155/2013/836989

**Published:** 2013-12-31

**Authors:** Lee D. Chaves, Liby Mathew, Mohammed Shakaib, Anthony Chang, Richard J. Quigg, Tipu S. Puri

**Affiliations:** ^1^Division of Nephrology, Department of Medicine, State University of New York at Buffalo School of Medicine and Biomedical Sciences, 875 Ellicott Street, Buffalo, NY 14202, USA; ^2^Section of Nephrology, Department of Medicine, The University of Chicago, 5841 South Maryland Avenue, Chicago, IL 60637, USA; ^3^Department of Pathology, The University of Chicago, 5841 South Maryland Avenue, Chicago, IL 60637, USA

## Abstract

Using a reversible UUO model (rUUO), we have demonstrated that C57BL/6 mice are susceptible to development of CKD after obstruction-mediated kidney injury while BALB/c mice are resistant. We hypothesized that selective systemic depletion of subpopulations of inflammatory cells during injury or repair might alter the development of CKD. To investigate the impact of modification of T_h_-lymphocytes or macrophage responses on development of CKD after rUUO, we used an anti-CD4 antibody (GK1.5) or liposomal clodronate to systemically deplete CD4^+^ T cells or monocyte/macrophages, respectively, prior to and throughout the rUUO protocol. Flow cytometry and immunohistochemistry confirmed depletion of target cell populations. C57BL/6 mice treated with the GK1.5 antibody to deplete CD4^+^ T cells had higher BUN levels and delayed recovery from rUUO. Treatment of C57BL/6 mice with liposomal clodronate to deplete monocyte/macrophages led to a relative protection from CKD as assessed by BUN values. Our results demonstrate that modulation of the inflammatory response during injury and repair altered the susceptibility of C57BL/6 mice to development of CKD in our rUUO model.

## 1. Introduction

Between 10 and 16% of the adult population worldwide is affected by chronic kidney disease (CKD) [1]. From the periods of 1988–1994 to 2005–2010 the prevalence of CKD in the United States rose from 12.3 to 14.0 percent. The largest relative increase, from 25.4 to 40.8 percent, was seen in those with cardiovascular disease [2]. The life expectancy for a 50-year-old adult in the United States is 35.5 years; this decreases by 7.5 years in the presence of CKD [2].

From among a variety of possible rodent models of CKD, the unilateral ureteral obstruction (UUO) model has become widely used to evaluate features of renal injury [3–5]. Advantages of the UUO model include the fact that kidney injury and fibrosis occur over a time course of days to weeks and that the model can be used in mice of any strain. Typically in the UUO model, obstruction is achieved by irreversible ligation of the ureter. Importantly, functional consequences of kidney injury cannot be assessed using irreversible obstruction and findings must be interpreted in the context of ongoing injury from obstruction.

To model CKD in mice we generated a reliable reversible UUO model (rUUO) [6]. This model combines several key advantages for studying development and progression of CKD, including assessment of functional consequences of kidney injury using biomarkers such as blood urea nitrogen (BUN) measurements and the ability to study pathophysiology during kidney injury and during recovery from injury. Our previous work with this model showed a distinct genetic basis for development of CKD and suggested an immunogenetic mechanism. In our model C57BL/6 mice, which generally produce a T helper cell type 1 immune response, progressed to CKD whereas BALB/c mice, with immune responses skewed to T helper type 2 responses, were resistant. Considering that, we hypothesize that one or more immune cell type may contribute to or be preventative of kidney disease development in our mouse model. To test that, in a separate set of experiments we systemically depleted CD4^+^ or monocyte/macrophage cells using an anti-CD4 antibody or liposome encapsulated clodronate, respectively, and subjected the mice to rUUO.

## 2. Materials and Methods

### 2.1. Murine RUUO Model

We used a murine rUUO model as previously described [6]. For these studies, 6–8 week male C57BL/6 mice had six days of UUO followed by release of obstruction. To do this, a microvascular clip was placed on the right ureter (day −6) and then adjusted distally after 2 days (day −4) and again after 4 days (Day −2). The clip was removed 6 days after the initial UUO. For consistency, the time when the ureteral obstruction was reversed will be termed day 0. Then, on day 7 (i.e., 13 days after UUO), the left kidney was removed. Thus, the animal relied solely upon function of previously obstructed kidney, which was quantified by measurement of blood urea nitrogen (BUN) levels. The use of animals in these studies was approved by the University of Chicago Institutional Animal Care and Use Committee.

### 2.2. Cell Depletions

CD4^+^ T cells were depleted in mice with monoclonal rat anti-mouse CD4 antibody GK1.5 (Fitch Monoclonal Antibody Facility, The University of Chicago). Briefly, intraperitoneal injection of 10 *μ*g/g body weight (~200 *μ*g) GK1.5 IgG or vehicle control was performed 1 day prior to and maintained during the rUUO protocol with three subsequent injections every 6 days (i.e., animals were injected on protocol days −7, −1, 5, and 11). CD4^+^ cellular depletion was confirmed by flow cytometry. 

Systemic monocyte/macrophage depletion was performed using clodronate liposomes (Encapsula NanoSciences). Empty liposomes in PBS were used as controls. Clodronate (200 *μ*g) or vehicle (PBS control) liposomes were administrated to mice intravenously starting 6 days prior to UUO and continuing every 3-4 days until 10 days after release of obstruction (i.e., animals were injected on protocol days −12, −8, −5, −1, 3, 6, and 10).

### 2.3. Histology

Tissue harvest: right kidneys were surgically removed under anesthesia. The kidneys were bisected through a coronal section and processed as follows for routine light microscopic evaluation. Pieces of renal tissue no more than 1–1.5 mm thick were fixed in 4% paraformaldehyde in PBS for 24 h at 4°C. Following fixation, tissue samples were routinely processed and embedded in paraffin wax (TissuePrep II, Fisher Scientific). Five 3 *μ*m tissue sections were cut onto Superfrost Plus slides (Fisher Scientific), dewaxed, and hydrated through a descending series of alcohols. 

Immunohistochemical staining was conducted using a modified Vectastain Elite ABC kit (Vector Laboratories). Briefly, sections were deparaffinized and endogenous peroxidase activity blocked by incubation in hydrogen peroxide. Epitope retrieval was carried out by microwave treatment (10 min on high setting), blocked for endogenous biotin and nonspecific background staining, and incubated with rabbit anti-mouse F4/80 antibody (sc-25830; Santa Cruz) or control IgG. Sections were washed in Tween 20-buffered saline (TBS) and incubated with a biotinylated anti-rabbit antibody. After washing with TBS, the sections were incubated with an avidin-horseradish peroxidase complex. Sections were rewashed and developed by diaminobenzidine tetrahydrochloride (Sigma-Aldrich). Procedures were carried out at room temperature unless otherwise noted. 

Tissue sections were stained by periodic acid-Schiff (PAS) and Masson's Trichrome using standard methods. Stained slides were reviewed by a renal pathologist (AC) for semiquantitative assessment of interstitial fibrosis and tubular atrophy (IF/TA) using the following 0–3 point scoring system: 0 ≤ 5% (none), 1 = 6–25%, 2 = 26–50%, and 3 ≥ 50%.

### 2.4. Flow Cytometry

Erythrocytes were removed from whole blood samples (50 *μ*L) with 4 mL erythrocyte lysing reagent (150 mM NH_4_Cl, 10 mM KHCO_3_, 0.5 M EDTA, pH 8) incubated at room temperature for 5 min. The process was repeated twice additionally, with the cells centrifuged at 250 g × 5 min prior to resuspension. Cells were then resuspended in 200 *μ*L PBS and incubated with anti-CD4 (GK1.5) for 20 min on ice, washed with 1 mL PBS and centrifuged as above, resuspended in 200 *μ*L PBS, and analyzed on a flow cytometer (FACScanto). Data were analyzed using FlowJo software (v. 10) and presented as percentage of peripheral blood mononuclear cells (PBMCs) that were CD4^+^.

Single-cell suspensions from whole kidneys at the indicated time were isolated. Whole kidneys were minced into small pieces (<1 mm) in ice-cold 1× HBSS media and incubated with 1 mg/mL collagenase (type IA, Sigma) and 0.1 mg/mL deoxyribonuclease (DNase, type I, Sigma) at 37°C for 25 min with gentle shaking. The suspension was centrifuged at 250 g × 5 min and the pellet was resuspended in 2 mL of erythrocyte lysing reagent and incubated for 5 min at room temperature. The suspension was again centrifuged at 250 g × 5 min, and the supernatant was discarded. The cells were resuspended in 1-2 mL of ice-cold PBS and passed through a 40 *μ*m cell strainer. Single-cell suspensions were incubated with anti-CD11b (M1/70) and F4/80 antibody (Cl : A3-1, Serotec). Cells were washed with ice-cold PBS (4 mL/wash) followed by centrifugation at 250 g × 5 min, resuspended in ice-cold PBS, and then analyzed on a flow cytometer (FACScanto). Data were analyzed using FlowJo software (v. 10).

### 2.5. Statistical Analyses

Numeric data were analyzed with Minitab software (v. 16.2.4). Data were confirmed to be normally distributed using Anderson-Darling tests. Comparisons between two groups of parametric data were made with two-sample *t*-testing. BUN and CD4^+^ cell data from all individual mice in the study are shown in the figures with means as horizontal lines. In the text, data are presented as means ± SEMs.

## 3. Results

### 3.1. Recovery from rUUO Is Delayed in Mice Depleted of CD4^+^ T Cells

**Figure 1:**
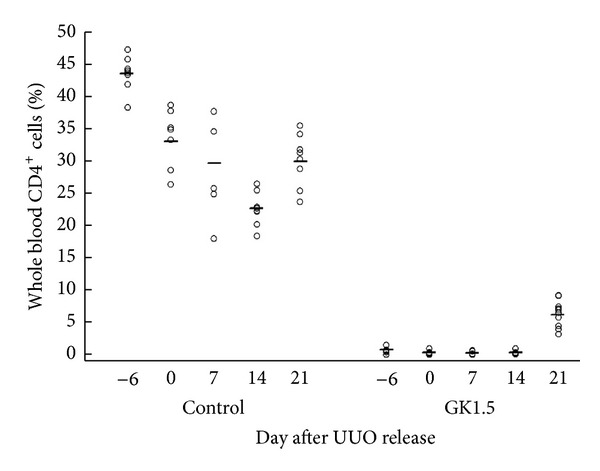
Flow cytometry of PBMCs confirming CD4^+^ depletion in mice treated with GK1.5.

In control mice subjected to the rUUO protocol, the percentage of CD4^+^ cells in total PBMCs declined (Figure 1). This was at least partially attributable to an expansion of non-CD4^+^ cells in the PBMC pool (i.e., rather than a decline in absolute CD4^+^ numbers). Anti-CD4 antibody treatment resulted in complete depletion of CD4^+^ T cells prior to UUO, which lasted through 14 days following release of obstruction (Figure 1). Of necessity we were limited to the number of GK1.5 injections and chose to span the 18 days beginning prior to UUO. Thus, because the final injection was on day 12 after release of UUO, there was evidence for reconstitution of CD4^+^ cells by day 21 after release of UUO (Figure 1).

**Figure 2:**
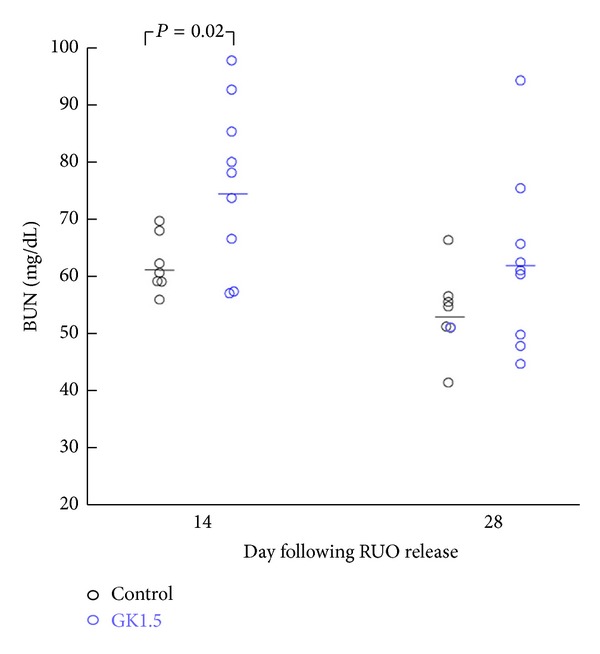
BUN measurements in CD4-depleted (GK1.5) and control mice on days 14 and 28 following release of UUO. On day 7, all animals had removal of the contralateral (unobstructed) kidney.

Our hypothesis was that CD4^+^ cells were involved in the pathophysiology of this rUUO model, either in the injury occurring during the period of UUO and/or after release. It was therefore surprising that BUN levels were significantly higher in CD4-depleted animals on day 14 compared to controls (Figure 2, 76.4 ± 4.8 versus 62.0 ± 1.9 mg/dL, resp.). As we typically see in this model of CKD, BUN levels are highest immediately after contralateral kidney removal and appear to equilibrate with time. Thus, as shown in Figure 2, BUN levels in control mice were lower on day 28 (53.8 ± 2.8 mg/dL). While BUN levels in CD4^+^ cell-depleted mice remained higher than controls (62.3 ± 5.1 mg/dL), these were not statistically different than controls at this time.

### 3.2. Depletion of Monocyte/Macrophages with Clodronate Attenuates CKD in rUUO

**Figure 3:**
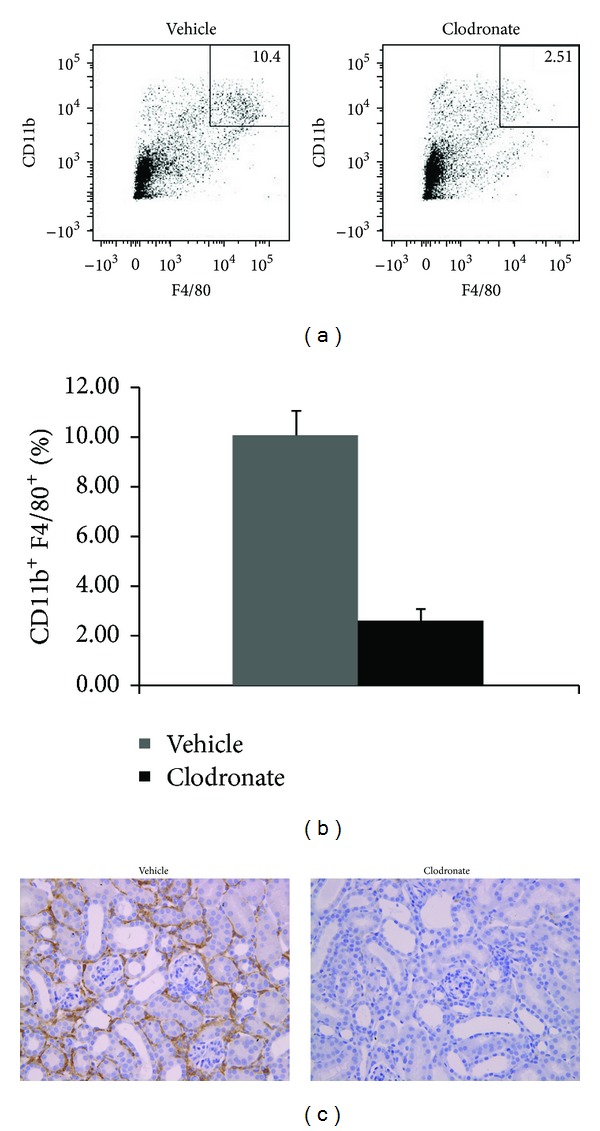
CD11b^+^ and F4/80^+^ cells in kidneys of clodronate-treated or control mice after 6 days of UUO (i.e., day 0 of the rUUO protocol). Representative flow cytometry is shown for CD11b^+^ and F4/80^+^ (a) with data from all kidneys shown graphically (b). Representative immunohistochemical staining for F4/80 is also shown (c). *N* = 5-6 per group.

**Figure 4:**
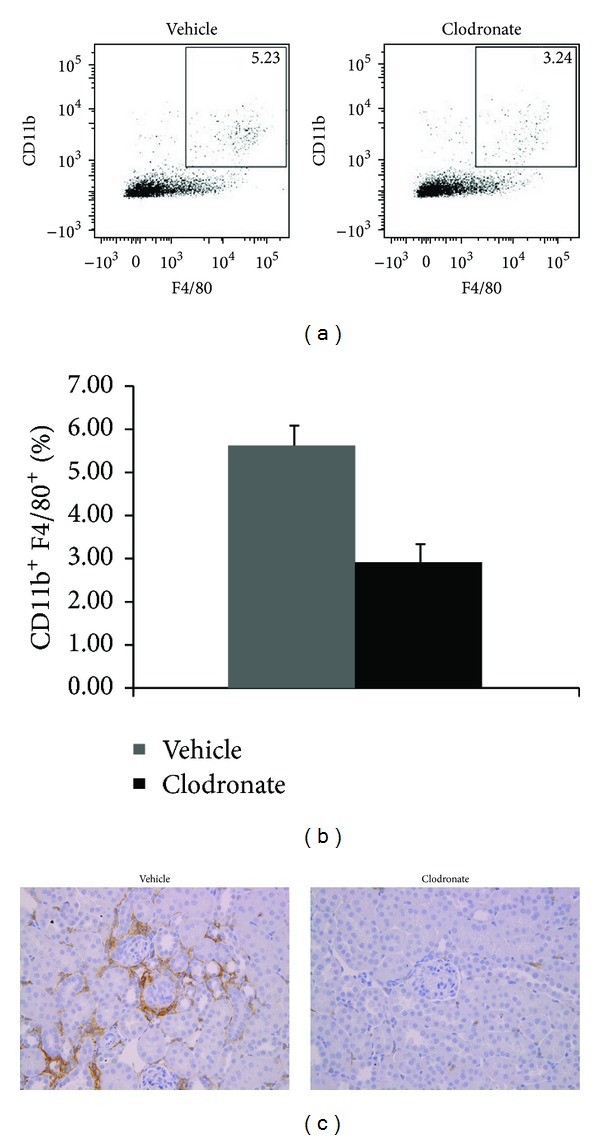
CD11b^+^ and F4/80^+^ cells in kidneys of clodronate-treated or vehicle-treated (control) mice 7 days after release of a 6 day UUO. Representative flow cytometry is shown for CD11b^+^ and F4/80^+^ (a) with data from all kidneys shown graphically (b). Representative immunohistochemical staining for F4/80 is also shown (c). *N* = 5 per group.

To evaluate mononuclear phagocytic cell effects in our rUUO model of CKD, clodronate or PBS control liposomes were administrated to mice intravenously. Kidneys were evaluated by flow cytometry and immunohistochemistry at days 0 and 7 after release of obstruction, while BUN levels were evaluated on Days 14 and 28 after release of obstruction. On day 0, kidney F4/80^+^CD11b^+^ cells were reduced by more than 75% (Figures 3(a) and 3(b)). Immunohistochemical staining of kidney sections confirmed the depletion (Figure 3(c)). At day 7, there was still nearly 50% less F4/80^+^CD11b^+^ cells in clodronate-treated mice compared to controls (Figures 4(a) and 4(b)).

**Figure 5:**
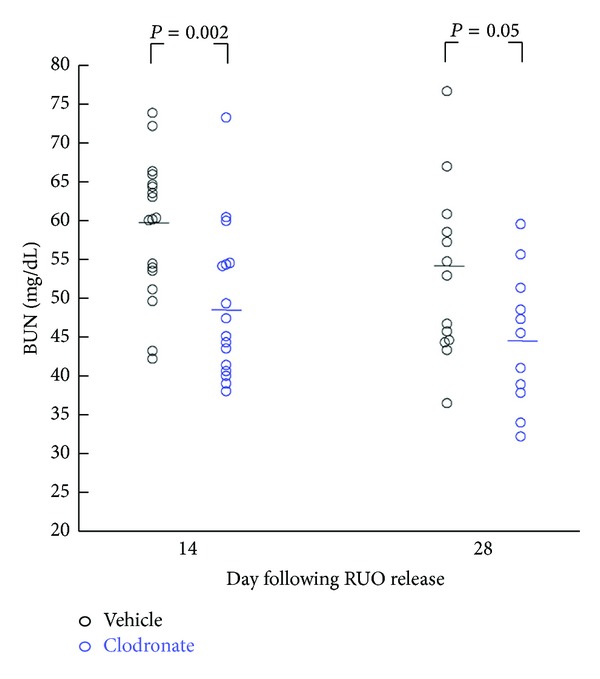
BUN measurements in clodronate-treated and vehicle-treated (control) mice on days 14 and 28 following release of UUO. On day 7, all animals had removal of the contralateral (unobstructed) kidney. *N* = 12–19 per group.

**Figure 6:**
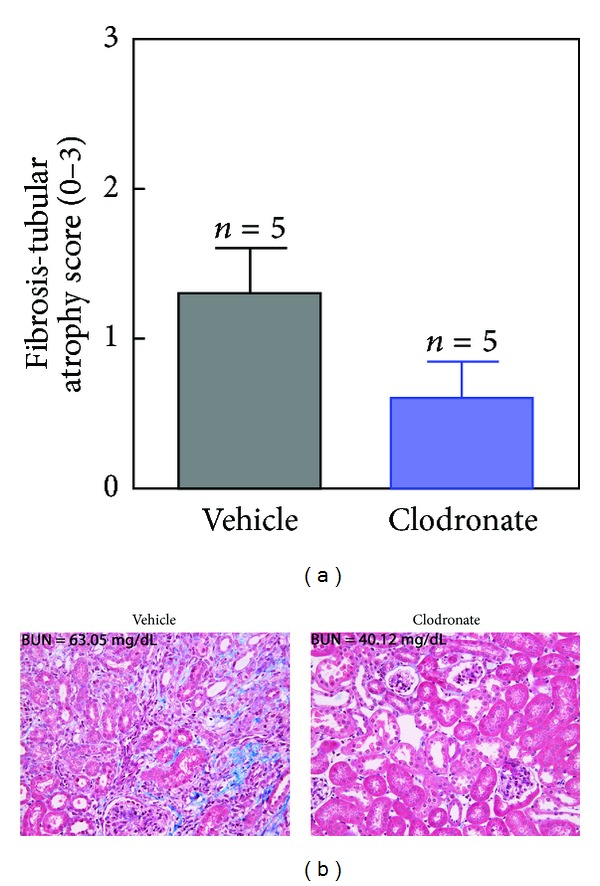
Interstitial fibrosis and tubular atrophy (IF/TA) scores at 14 days following release of UUO in vehicle- (black) and clodronate- (blue) treated mice (5 mice per treatment group, Y-error bars indicate SEM) (a). Representative Masson's Trichrome staining is shown with the BUN for the representative animals (b).

Depletion of mononuclear cells with clodronate led to a relative protection from CKD as assessed by BUN values (Figure 5). Thus, 14 days after release of obstruction, BUN values in control and clodronate-treated mice were 59.1 ± 2.1 and 48.6 ± 2.3 mg/dL. Semiquantitative assessment of interstitial fibrosis and tubular atrophy (IF/TA) based on PAS and Masson's Trichrome staining was performed by a renal pathologist blinded to the origin (treatment group) of slides using a 0–3 scale (as defined in the Methods). Vehicle-treated mice had a higher fibrosis score as compared to clodronate-treated mice (1.3 ± 0.30 versus 0.6 ± 0.24, mean ± SEM) at 14 days after release of obstruction (Figure 6(a)). IF/TA scores were consistent with the lower BUN levels in clodronate-treated mice after rUUO. Representative Masson's Trichrome stained sections (Figure 6(b)) illustrate the histological differences between the two treatment groups. Despite the fact that clodronate was not administered to animals after day 10, this relative protection was maintained through day 28. BUN values were 53.1 ± 3.1 and 44.8 ± 2.6 mg/dL in control and clodronate-treated mice, respectively (Figure 5).

## 4. Discussion

The functional development of CKD is characterized by histopathological features of renal parenchymal loss and replacement by fibrotic tissue. UUO is commonly used to induce renal fibrosis in rodents; as such, it is considered to be a viable model of human CKD [3–5]. Significant efforts have focused on characterizing responses to kidney injury, such as occurs in UUO, by inflammatory cells, growth factors, cytokines, matrix proteins, and other mediators [5, 7, 8]. Yet, in human renal diseases, periods of repair, including those induced by therapeutic maneuvers, often intervene. By allowing the study of responses related to both injury and repair, our model of rUUO provides considerable pathophysiological relevance.

Activation of profibrotic pathways, such as the transforming growth factor-*β* pathway, triggers profibrotic events including transcription of matrix protein genes and factors involved in matrix metabolism [9, 10]. Other mediators such as angiotensin II, connective tissue growth factor, and platelet derived growth factor have also been implicated in development of renal fibrosis and progression of kidney disease [11, 12]. Myofibroblasts are considered to be the primary source of the interstitial collagen contributing to fibrosis, including that seen in UUO models. The exact origin of the myofibroblast, from among local resident pericytes and/or fibroblasts, bone marrow-derived cells, and/or via epithelial- and endothelial-to-mesenchymal transition, continues to be debated [11, 13, 14]. In recent studies from Raghu Kalluri's group using the UUO model, the proportions of the latter three were approximately 50, 35, and 15%, respectively [15], while pericytes did not give rise to myofibroblasts. The latter is in distinction to work from Duffield's group showing a prominent role for pericyte-derived myofibroblasts [16, 17].

It is widely believed that CD4^+^ cells affect the differentiation of monocytes into fibrocytes in chronic disease models [18]. In C57Bl/6 mice undergoing the 7-day UUO model, depletion of CD4^+^ cells with GK1.5 led to ~20% reduction in collagen I deposition [19]. An additional study in C57Bl/6 mice of a 14-day UUO model utilizing the monoclonal anti-CD4 YTA3.1 for cell depletion found a similar 20% reduction in collagen deposition [20]. The authors further showed that RAG-/- mice developed significantly less collagen deposition in their 14-day UUO model and reconstitution of RAG-/- mice with CD4^+^ cells, but not CD8^+^ cells 14 days prior to UUO resulted in collagen deposition comparable to wild type mice. In contrast, our data in the model of rUUO in C57Bl/6 suggest that CD4^+^ T cells or a subpopulation thereof (e.g., T_h_1 or T_reg_) may have a protective role in the early stages of kidney disease development; however, any such benefit is overcome in the later stages of disease. Targeting a specific subpopulation in future studies may better define the role(s) of CD4^+^ cells. In nonreversible UUO models others have described evidence suggesting a role for CD4^+^ cells in the development of renal fibrosis. In recent studies in BALB/c mice, Liu et al. demonstrated that CD4^+^ depletion caused a reduction in renal fibrosis due to ureteral obstruction [21]. In a nude mouse T cell reconstitution experiment they provided further evidence suggesting that the fibrosis can be attributed to T_h_2 subsets. We previously demonstrated in our reversible obstruction model that BALB/c mice, genetically skewed to T_h_2 responses, were resistant to the development of CKD [6]. The seemingly contradictory results may be explained by the absence of regulatory T cells in studies with nude mice. Perhaps the T_h_2 T cell phenotype, through their production of IL4, promotes M2 macrophage skewing *in vivo*. M2 macrophages are thought to promote healing while limiting fibrosis and can serve as antigen presenting cells for the activation and propagation of T_h_2 and T_reg_ cell responses [22]. Ureteral obstruction models are sterile, pathogen-free diseases. Interestingly, the cognate antigen of most regulatory T cells is a “self-antigen,” which may underscore the necessity of this population to prevent fibrosis in the wild type mouse.

In a nonreversible model of ureteral obstruction, Kitamoto et al. utilized clodronate liposomes to evaluate renal phagocytes [23]. They obtained a similar level of F4 80^+^ cell depletion and, interestingly, a reduction of renal fibrosis as measured by collagen II and smooth muscle actin deposits in the interstitium. These results appear to coincide with our function data showing a diminished extent of kidney damage as measured by BUN in clodronate-treated mice undergoing reversible ureteral obstruction. The same group followed up by selectively depleting either CD11b or CD11c expressing cells [24]. Systemic depletion of CD11c expressing cells had no effect on the development of renal fibrosis in their ureteral obstruction model. Alternatively, systemic depletion of CD11b expressing cells prior to obstruction resulted in a significant reduction of fibrosis markers in renal tissue. However, the role of either cell population in the recovery and further kidney function after obstruction reversal is still undefined. Further confounding is the nearly ubiquitous expression pattern of CD11b in the myeloid cell lineage. For example, Summers et al. provided evidence suggesting a role of mast cells, which also express CD11b, in initial renal fibrosis development due to ureteral obstruction [25].

Chronic kidney disease afflicts over 10% of the population over 20 years of age and more than 40% of the population age 65 and over. Understanding both the physiological and cellular processes that contribute is essential in order to mitigate or even prevent progression into end stage renal disease. By using our rUUO model of CKD in lieu of standard UUO models we can study the cellular mechanisms that lead to functionally significant kidney damage. Our future studies are designed to further examine these cellular responses including the likely possibility that there is interdependence in responses by different cell populations and subsets. Through this work we hope to elucidate the roles and mechanisms by which lymphocyte and mononuclear phagocyte subsets contribute to fibrosis and repair, for example, T_h_2, T_reg_ or M1, M2 macrophages, respectively.
